# RAS-Selective Lethal 3-Induced Ferroptosis Promotes the Antitumor Efficiency of Anti-Programmed Cell Death Protein 1 Treatment in Colorectal Cancer

**DOI:** 10.5152/tjg.2023.23300

**Published:** 2024-04-01

**Authors:** Shiyv Lu, Zhilu Yao, Qing Cheng, Jianping Wu, Yuanye Jiang, Hui Lin

**Affiliations:** 1 Department of Gastroenterology, Shanghai Jing’an District Zhabei Central Hospital, Shanghai, China; 2 Department of Gastroenterology, Shanghai Qingpu District Hospital of Traditional Chinese Medicine, Shanghai, China; 3 Department of Gastroenterology, Putuo Hospital, Shanghai University of Traditional Chinese Medicine, Shanghai, China

**Keywords:** Ferroptosis, anti-PD-1, colorectal cancer, CD8+ T cells

## Abstract

**Background/Aims:**

Anti-programmed cell death protein 1 (PD-1) treatment has exhibited clinical benefits in colorectal cancer (CRC). However, the low response rate of CRC to immunotherapy is an urgent problem that needs to be solved.

**Materials and Methods:**

MC-38 tumor cells was challenged subcutaneously in the flank of 7-week-old male C57BL/6 mice. The mice were randomly divided into 3 groups, and 200 µg/mouse anti-PD-1 antibody and 100 mg/kg RAS-Seletive Lethal 3 (RSL) or phosphate buffer saline (PBS) were intraperitoneally injected every 2 days. The expression of oxidative stress and ferroptosis-related genes was measured by Western blotting, real-time reverse transcription-polymerase chain reaction, Prussian blue staining, and enzyme-linked immunosorbent assay.

**Results:**

Anti-PD-1 treatment-unresponsive tumors showed stronger immunosuppression than responsive tumors. Notably, the responsive tumors showed higher levels of H_2_O_2_ and reactive oxygen species, both of which could impair the antitumor effect of cytotoxic CD8^+^ T cells. The anti-PD-1 treatment-responsive tumors showed a higher expression of pro-ferroptosis genes and Fe^2+^ accumulation than those of anti-PD-1 nonresponsive tumors, indicating the potential role of ferroptosis in the efficacy of anti-PD-1 treatment. In MC-38 syngeneic tumor model, (1S, 3R)‐RSL3 (RSL), a glutathione peroxidase 4 inhibitor, effectively promoted the antitumor effect of anti-PD-1 treatment in vivo. However, anti-PD-1 treatment did not affect the levels of ferroptosis-related genes in tumor model. Mechanistically, RSL treatment significantly upregulated the frequency of proliferating (ki67^+^) and cytotoxic (GZMB^+^) CD8^+^ T cells. Furthermore, the frequency of tumor neoantigen-specific interferon (IFN)-γ CD8^+^ T cells showed a significant increase after RSL plus anti-PD-1 treatment.

**Conclusion:**

RSL may be a promising drug for potentiating the antitumor efficiency of anti-PD-1 treatment in CRC.

**Main Points**

- Intratumor ferroptosis promotes anti-programmed cell death protein 1 (PD-1) therapy for colorectal cancer (CRC).
- Ferroptosis enriches the tumor neoantigen-specific CD8^+^ T cells repertoire.
- Combining anti-PD-1 and pro-ferroptosis therapy can inhibit CRC progression.

## INTRODUCTION

Colorectal cancer (CRC) is one of the leading causes of cancer-related death; more than 945 000 people develop CRC worldwide, and approximately 50% of them die every year.^1,2^ Among people who are diagnosed with metastatic CRC, less than 20% survive beyond 3 years.^3^ Traditionally, the common treatments for unresectable metastatic CRC include cytotoxic chemotherapy and radiotherapy.^3^ However, the strong side effects due to their nonspecificity and cytotoxicity towards proliferating cells and the availability of somatic variants severely hindered the effectiveness of chemotherapy and radiotherapy. Therefore, a novel and better strategy urgently needs to be developed.

Recently, immunotherapy targeting epidermal growth factor receptor (EGFR) and immune checkpoints has aroused wide concern about CRC therapy.^4-6^ A clinical trial demonstrated that approximately 50% of metastatic CRC patients who received EGFR monoclonal antibodies and chemotherapy survived 2-4 months longer than those who received chemotherapy alone. However, this treatment could only be adopted for patients with EGFR mutations.^7,8^ Therefore, immunotherapy targeting immune checkpoints, such as programmed cell death protein 1 (PD-1) and programmed death-ligand 1 (PD-L1) immunotherapy, has been widely studied in multiple tumors. In CRC, anti-PD-1 (pembrolizumab and nivolumab) and anti-CTLA-4 inhibitors (nivolumab) were approved by the FDA, and their treatment efficiency is influenced by the microsatellite instability and tumor mutation environment in each CRC patient.^9-11^ A total of 69% of patients treated with anti-PD-1 inhibitors have 12 months of overall survival after pembrolizumab and nivolumab treatment.^12^ Interestingly, a combination of nivolumab with ipilimumab could efficiently improve the therapeutic effect.^13^ Even so, a large number of CRC patients are not responsive to anti-PD-1 and anti-CTLA-4 treatment. Therefore, we would like to discover an effective auxiliary strategy to further enhance the efficiency of immunotherapy.

Previous studies have shown that cell death induced by ferroptosis is a critical event and participates in malignant tumor development.^14,15^ Ferroptosis is a novel form of regulated cell death that is a different cell death model for apoptosis and necrosis and is characterized by mitochondrial abnormalities and iron-dependent lethal lipid peroxide accumulation.^16^ Ferroptosis is an iron-dependent cell death, and it has been implicated in various human diseases, including CRC.^17,18^ Compared to apoptosis, there is evidence that ferroptosis appears to be more immunogenic due to the release of damage-associated molecular patterns, which in turn exacerbate the inflammatory response by releasing many more tumor-specific neoantigens,^19^ which are subsequently recognized and captured by major histocompatibility complex I (MHC-I) and T-cell receptor (TCR).^20^ In contrast, it was also reported that the ferroptosis of CD8^+^ T cells undermined the potent antitumor effects, indicating the complicated relationship between ferroptosis and tumor immunotherapy.^21^ However, a clear effect of ferroptosis on anti-PD-1-mediated immunotherapy remains to be explored in CRC.

Here, we hypothesized that ferroptosis-induced death of tumor cells could enhance the emission of tumor-derived peptides, named tumor-specific neoantigens, which can strongly increase the population of tumor-specific CD8^+^ T cells. Then, these expanded tumor-specific CD8^+^ T cells were maintained with strong antitumor effects after anti-PD-1 treatment. In this study, we analyzed the status of ferroptosis in anti-PD-1 responsive and unresponsive MC-38 tumor samples and explored the effect and underlying mechanism of the ferroptosis inducer, RSL, on the antitumor effect of anti-PD-1 antibody on syngeneic CRC mouse model.

## MATERIALS AND METHODS

### Cell Culture

The murine colon adenocarcinoma cell line MC-38 cell line was purchased from American Type Culture Collection (ATCC) and cultured in 1640 medium supplemented with 10% fetal bovine serum (FBS) and 1% antibiotics at 37 °C in a humidified 5% CO_2_ incubator.

### Tumor Model

Seven-week-old male C57BL/6 mice (20 ± 1 g) were purchased from Nanjing Model Animal Center (Nanjing, China). All animals were housed in a controlled room at 23 ± 2°C, 65%-75% humidity, and a 12-hour light–dark cycle. Water and commercial laboratory-complete animal chow are available. The logarithmic growing MC-38 cells were collected and resuspended in 150 μL of cold PBS. Then, 2 × 10^6^ cells/mouse were injected into the flanks of the mice. Seven days later, the mice were randomly divided into 4 groups, and each mouse was treated with 200 µg/mouse of PBS or anti-PD-1 treatment or 100 mg/kg RSL every 2 days. The tumor volume at different timepoints was measured by caliper, and the calculation formula was (length × width × width) × 0.5. The mice were sacrificed at the endpoint, and the tumors were weighed by an analytical balance. All animals received humane care according to the criteria outlined in the ‘‘Guide for the Care and Use of Laboratory Animals’’ prepared by the National Academy of Sciences and published by the National Institutes of Health. All procedures were approved by the Animal Ethics Committee of Putuo Hospital. This study was approved by the Ethics Committee of Shanghai University of Chinese Tranditional Medicine (Approval No: DWEC-A-202206013M, Date: 2022-06-01).

### Reverse Transcription Polymerase Chain Reaction

Total messenger RNA (mRNA) was extracted by TRIzol reagent, and complementary DNA (cDNA) was obtained by reverse transcription of 1 μg of mRNA with the Go ScriptTM Reverse Transcription System. The cDNA was used as the template after 5 dilutions. SYBR green was used to quantify the expression of genes. The relative expression of targeted genes was normalized to glyceraldehyde 3-phosphate dehydrogenase. The relative quantification of gene expression was analyzed using the 2^–ΔΔCt^ method. Three replicates were included for each sample.

### Western Blot

After the tumor tissues were isolated, protein was extracted by radioimmunoprecipitation lysis buffer according to an established protocol. Protein concentration was determined using the bicinchoninic acid protein assay. Protein extracts were electrophoresed in 10% sodium dodecyl-sulfate polyacrylamide gel electrophoresis (SDS‒PAGE), transferred to nitrocellulose filters, and blocked with 5% skim milk for 1 hour at room temperature. The membranes were then immunoblotted with targeted primary antibodies overnight at 4°C. The membranes were washed with tris buffered saline with tween (TBST) 3 times and incubated with secondary antibody for 1 hour at room temperature. Then, the blots were exposed and analyzed by using ImageJ.

### Flow Cytometry

Cells were resuspended in PBS containing 1% FBS and blocked with Fc block for 10 minutes. Five microliters of fluorescence-conjugated primary antibody (anti-CD3, anti-CD4, anti-CD8, anti-GZMB, or anti-Ki67) was added and incubated for 30 minutes on ice in the dark. Then, the cells were washed with cold PBS 3 times in the dark. The supernatant was removed after centrifugation at 350 g, and the cell pellet was resuspended in cold PBS for analysis on a Fortessa. All files were calculated by Flow Jo software.

### Reactive Oxygen Species Detection

Reactive oxygen species (ROS) levels in tumor resuspension were measured using a 2,7-dichlorodihydrofluorescein diacetate ROS assay kit according to the manufacturer’s instructions. Briefly, the samples were prepared and then incubated with 2,7-dichlorodihydrofluorescein diacetate at 37°C in darkness. Afterward, the cells were resuspended in fresh RPMI 1640 medium, and the signals were photographed under a fluorescence microscope.

### H_2_O_2_ Detection

Amplex Red kits were used to measure the level of H_2_O_2_ in the cell lysate according to the manufacturer’s instructions. Briefly, Amplex Red is a fluorescent probe highly sensitive to hydrogen peroxide and peroxidase. In the presence of peroxidase, Amplex Red can react with H_2_O_2_ 1 : 1 to produce strong red fluorescence. After the reaction, the absorbance was measured at A570, and the level of H_2_O_2_ was calculated according to the standard curve.

### Immunohistochemistry and Hematoxylin and Eosin 

The tumors were harvested, and serial tissue sections were subjected to hematoxylin and eosin (H & E) staining and immunohistochemistry. Briefly, the tissue was fixed with 4% paraformaldehyde, and 5 μm sections were produced. After antigen renaturation and primary antibody and secondary antibody incubation, the sections were stained with diaminobenzidine (DAB) and hematoxylin. Finally, the sections were sealed with neutral resin and scanned under a microscope.

### ELISpot Assay

ELISpot assays were performed on freshly isolated tumor-infiltrating CD8^+^ T cells. Assays were performed using Multiscreen IP ELISpot plates and coated with 20 μg/mL of mouse anti-IFN-γ coating antibody overnight at 4°C. Then, 1 × 10^5^ CD8^+^ T cells were added to each well of the plate, along with 10 µg/mL peptide pools covering key mutations in the MC-38 cell genome. After washing 5 times, 1 μg mL/1 anti-IFN-γ detection antibody was added. Alkaline phosphatase labelled SA (SA-ALP) (1 : 1000) was added after 4 hours of incubation. Add for 1-2 hours. Finally, the plate was developed under BCIP NBT-plus chromogenic substrate. The number of IFN-γ enzyme-linked immunosorbent assay spots was counted and analyzed.

### Isolation of Tumor-Infiltrating CD8^+^ T Cells

Tumors were digested with collagenase and hyaluronic acid, and 1 mL single-cell suspensions containing 1 × 10^8^ cells/mL were prepared for CD8^+^ T-cell isolation. The cell suspensions were blocked with normal rabbit serum and incubated with a cocktail for 10 minutes. Then, the beads targeted to CD8 T cells were added and mixed sufficiently for incubation at room temperature for 5 minutes. The total volume was diluted to 2.5 mL and placed on a magnetic pole for 2 minutes. Finally, the supernatant containing CD8^+^ T cells was collected and centrifuged at 350 g for 5 minutes.

### Coculture of Tumor Cells and CD8^+^ T Cells

Tumor-infiltrating CD8^+^ T cells were isolated as described above, and 96-well plates were precoated with 1 μg/mL anti-mouse CD3 overnight at 4°C. CD8^+^ T cells were labeled with 5 μM carboxyfluorescein diacetate succinimide ester (CFSE) for 20 minutes in an incubator. Labeled cells were washed with cold PBS once to remove excessive CFSE. Then, 0.05 M β-mercaptoethanol and 2 μg/mL anti-mouse CD28 were added to stimulate and activate CD8^+^ T cells. Tumor cells and CD8^+^ T cells were seeded into wells and incubated at 37 °C in a 5% CO_2_ incubator for 3 days. Finally, CFSE signals were detected by flow cytometry.

### Statistical Analysis

All data are presented as the mean ± standard error of mean. Analysis of variance (ANOVA) for different groups (same experiment) was performed using 1-way ANOVA and Tukey’s multiple comparison test. Unpaired Student’s *t* tests were adopted for comparisons between 2 groups. All statistical analyses were performed with GraphPad Prism 5.0 software.

## RESULTS

Anti-PD-1-responsive MC-38 tumors showed weak immunosuppression and strong oxidative stress.

**Figure 1.**
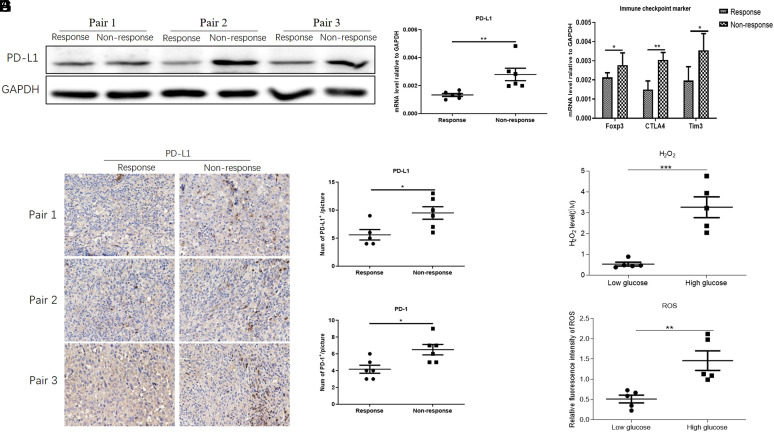
Anti-PD-1 treatment-responsive CRC tumors showed weak immunosuppression and strong oxidative stress. (A) The expression of PD-L1 in responsive and nonresponsive CRC tumors. GAPDH was used as the loading control. (B) The mRNA level of PD-L1 in responsive and nonresponsive CRC tumors, n = 5. (C) Detection of PD-L1 expression in 3 pairs of responsive and nonresponsive tumors by IHC. The statistics of (D) PD-L1^+^ and (E) PD-1^+^ cells evaluated by IHC in Figure 1C. Image-Pro 5.0 was used to count PD-L1^+^ cells (F) The mRNA levels of the immunosuppressive markers FOXP3, CTLA4, and Tim3 in anti-PD-1 treatment-responsive and nonresponsive tumors, n = 5. The expression was normalized to the expression of GAPDH. The levels of (G) H_2_O_2_ and (H) ROS in responsive and nonresponsive tumors. CRC, colorectal cancer; GAPDH, glyceraldehyde 3-phosphate dehydrogenase; IHC, immunohistochemistry; mRNA, messenger RNA; PD-1, programmed cell death protein 1; ROS, reactive oxygen species.

To investigate the mechanism underlying the unresponsiveness of MC-38 tumors to PD-1/L1 inhibitors, a syngeneic MC-38 mouse model was constructed, and anti-PD-1 was administered. The tumors were divided into anti-PD-1-responsive and nonresponsive cohorts according to the Response Evaluation Criteria in Solid Tumors guidelines. In total, 55% of mice were unresponsive to PD-1 administration, and 25% of tumors did not progress after 4 dosages of PD-1 treatment. Only 20% of tumors presented a significant decrease in volume and weight. PD-1-responsive and PD-1-unresponsive tumors were collected to explore the intratumor immune status of MC-38 cells. Considering the key roles of the PD-1/PD-L1 immune checkpoint in immunosuppression, we first measured the expression level of PD-1/L1 in MC-38 tumors. The Western blot results showed a significant increase in PD-1 and PD-L1 levels in responsive tumors compared with those in corresponding unresponsive tumors (Figure 1A). Real-time polymerase chain reaction (PCR) also confirmed the upregulation of PD-L1 at the transcriptional level in tumors (Figure 1B). In addition, we measured the PD-1 and PD-L1 expression levels by IHC in MC-38 tumor samples. Consistently, we observed a significant increase in PD-1- and PD-L1-positive cells in responsive tumors compared to those in unresponsive tumors (Figure 1C-E). Finally, we also analyzed the expression levels of 3 other common immunosuppression markers, Foxp3, CTLA4, and Tim3, in MC-38 samples. We found that all the unresponsive tumors showed higher levels of Foxp3, CTLA4, and Tim3 than responsive tumor tissues (Figure 1F). Paradoxically, we surprisingly found that the responsive tumors showed higher levels of H_2_O_2_ and ROS (Figure 1G-1H), both of which impaired the antitumor effect of cytotoxic CD8^+^ T cells by oxidative stress in previous studies. Overall, the oxidative stress in responsive tumors might contribute to the stronger antitumor effect of anti-PD-1 in a new manner.

The response of CRC to anti-PD-1 treatment is associated with markers of ferroptosis in anti-PD-1-responsive tumors.

**Figure 2.**
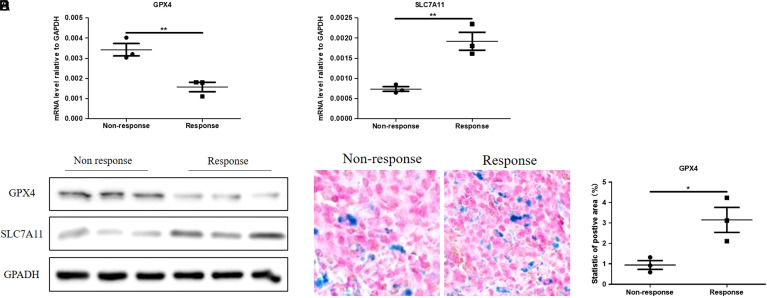
The response of CRC tumors to PD-1 Abs is associated with markers of ferroptosis in clinical samples. (A) The mRNA level of GPX4 in anti-PD-1 treatment-responsive and nonresponsive tumors. (B) The mRNA level of SLC7A11 in anti-PD-1 treatment-responsive and nonresponsive tumors. (C) The protein levels of GPX4 and SLC7A11 in anti-PD-1 treatment-responsive and nonresponsive tumors. (D) Prussian blue staining of anti-PD-1 treatment-responsive and nonresponsive tumors. (E) Statistics of Prussia blue staining. Image-Pro 6.0 was used to calculate the positive signal area.CRC, colorectal cancer; GPX4, glutathione peroxidase 4; mRNA, messenger RNA; PD-1, programmed cell death protein 1.

The antitumor effect of CD8^+^ T cells is mainly regulated by self-immunosuppression mediated by immune checkpoints and tumor-specific antigen stimulation. Considering the blockade of immune checkpoints in the study, we propose that the increased tumor-specific antigen presentation may explain the efficient antitumor effect of anti-PD-1-responsive tumors. Accumulating studies have reported a close correlation between oxidative stress and ferroptosis.^22-24^ Therefore, we investigated the level of ferroptosis in MC-38 tumors. We found that 2 crucial ferroptosis-promoting genes, GPX4 and SLC7A11, were significantly decreased in anti-PD-1-responsive tumors at both the mRNA and protein levels (Figure 2A-2C), indicating a stronger level of ferroptosis than that in nonresponsive tumors. Prussian blue staining showed the strong accumulation of iron in anti-PD-1-responsive MC-38 tumors (Figure 2D-2E). Consistently, the genes promoting ferroptosis in responsive tumors were significantly higher than those in unresponsive tumors. In conclusion, we inferred that a higher level of ferroptosis promotes the antitumor effect of anti-PD-1 in MC-38 tumors.

RSL-induced ferroptosis promoted the antitumor effect of anti-PD-1 in a syngeneic tumor mouse model.

**Figure 3.**
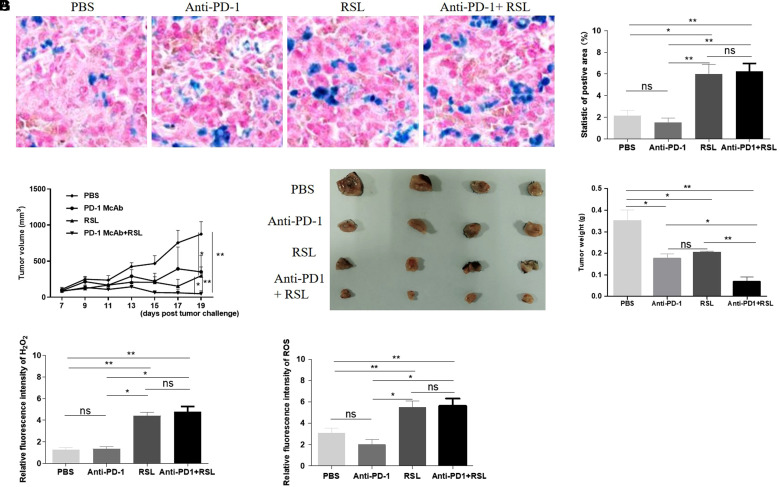
RSL-induced ferroptosis promoted the antitumor effect of anti-PD-1 in a syngeneic tumor mouse model. (A) Prussian blue staining of the tumor tissue of the PBS, anti-PD-1, RSL, and RSL+ anti-PD-1 treatment groups, n = 3. (B) The statistics of (D). Image-Pro 6.0 was used to calculate the Prussian blue-positive area. (C) The tumor growth rate in the MC-38 subcutaneous tumor model of different groups. The mice were treated with PBS, anti-PD-1, RSL, and RSL+ anti-PD-1. The tumor volumes were measured with callipers. The calculation formula is ½ (length × width × width). (D and E) The tumor weight at the endpoint in different groups, n = 5. (F and G). The levels of H_2_O_2_ and ROS in the syngeneic tumor mouse model treated with PBS, anti-PD-1, RSL, and RSL+ anti-PD-1. PD-1, programmed cell death protein 1.

To demonstrate whether enhanced ferroptosis could enhance the antitumor effect of anti-PD-1 in vivo, RSL was administered to a syngeneic MC-38 tumor model. The mice were randomly divided into 4 groups after tumor challenge, which were treated with PBS, anti-PD-1, RSL and RSL+ anti-PD-1. We measured the tumor volume at different timepoints during the process of different treatments. As expected, the results of Prussian blue staining showed stronger ferroptosis after RSL administration (Figure 3A-3B). We found that RSL+ PD-1 anti-treatment exhibited a stronger effect on the tumor growth rate than PBS and RSL or anti-PD-1 alone (Figure 3C). In addition, our results also demonstrated that RSL + anti-PD-1 therapy can efficiently decrease the volume and weight of tumors compared with those of either RSL or anti-PD-1 treatment alone, indicating the auxiliary effect of RSL on the antitumor effect of anti-PD-1 treatment in a syngeneic MC-38 tumor mouse model (Figure 3D-3E). Since the upregulation of oxidative stress induced by peroxides, such as ROS and H_2_O_2_, is one of the crucial markers of ferroptosis, we measured the level of ROS in the tumor lysates of all 4 groups. The results showed that RSL treatment significantly upregulated the H_2_O_2 _and ROS levels in tumors compared with the PBS- and anti-PD-1-treated groups, while no difference between the RSL- and anti-PD-1-treated groups was observed (Figure 3F-3G).

Anti-PD-1 treatment did not affect the levels of ferroptosis-related genes in tumors in a mouse model.

**Figure 4.**
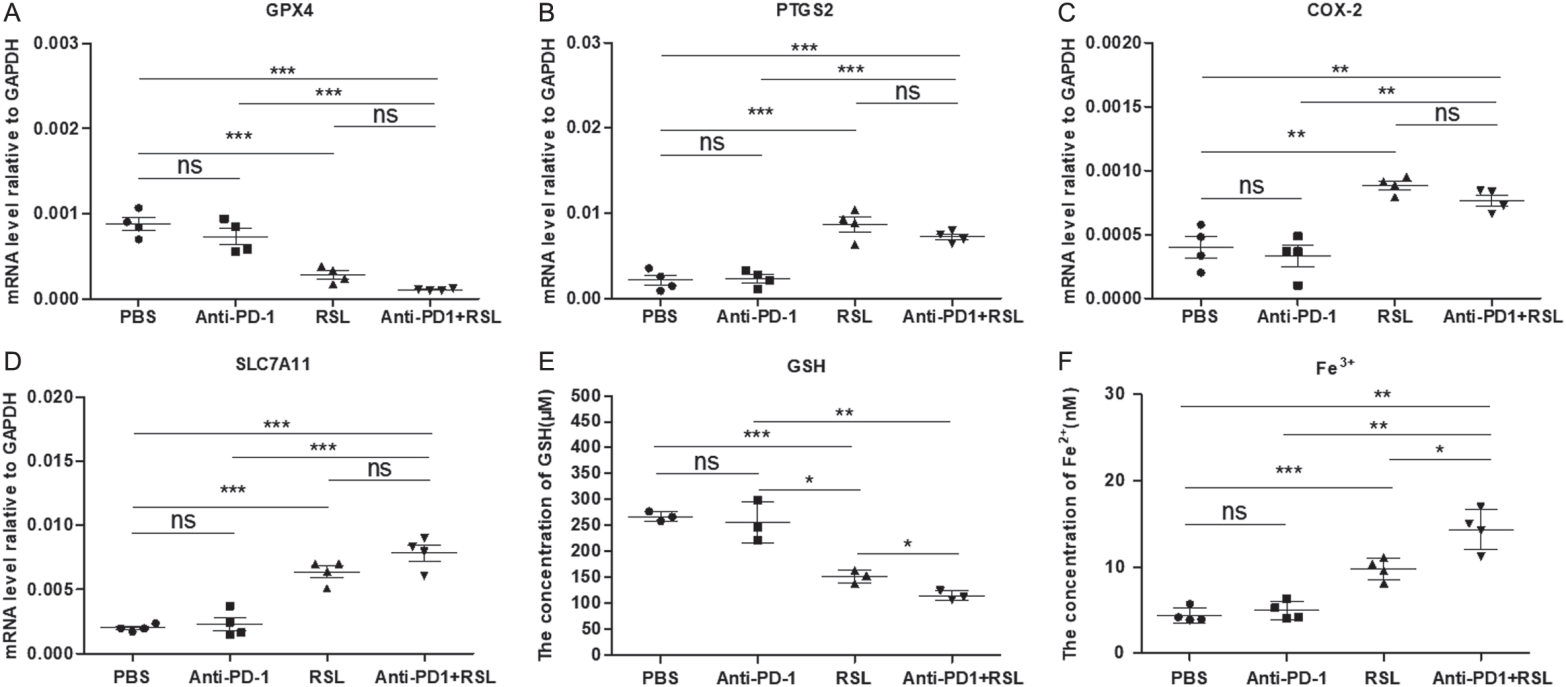
Anti-PD-1 treatment did not affect the levels of ferroptosis-related genes in tumors in a mouse model. (A–D) The mRNA levels of GPX4, PTGS2, COX-2, and SLC7A11 in the PBS, anti-PD-1, RSL, and RSL+ anti-PD-1 treatment groups, n = 4. (E) The level of GSH in the tumor suspension in the PBS, anti-PD-1, RSL, and RSL+ anti-PD-1 treatment groups, n = 4. (F) The level of Fe^2+^ in the tumor suspension in the PBS, anti-PD-1, RSL, and RSL+ anti-PD-1 treatment groups, n = 4. GPX4, glutathione peroxidase 4; mRNA, messenger RNA; PD-1, programmed cell death protein 1.

To further investigate whether anti-PD-1 treatment alone affected the level of ferroptosis in tumors, we analyzed the changes in ferroptosis-related factors, including GPX4, PTGS2, SLC7A11, COX-2, GSH, and Fe^2+^, in tumors from different groups. Real-time PCR results showed that RSL treatment significantly upregulated the levels of GPX4, PTGS2, COX-2, and SLC7A11, whereas anti-PD-1 treatment alone did not significantly change these genes (Figure 4A-D). In addition, we analyzed the levels of GSH and Fe^2+^ in different groups, which reflected the oxidative stress level and the accumulation of Fe^2+^ for ferroptosis. However, PD-1 treatment further decreased the level of GSH and upregulated Fe^2+^ levels when combined with RSL (Figure 4E and 4F). Therefore, PD-1 treatment did not affect the levels of ferroptosis-related genes but regulated the levels of GSH and Fe^2+^ in tumors in a syngeneic mouse model.

RSL may promote the antitumor effect of anti-PD-1 treatment by increasing the proliferation and cytotoxicity of CD8^+^ T cells.

**Figure 5.**
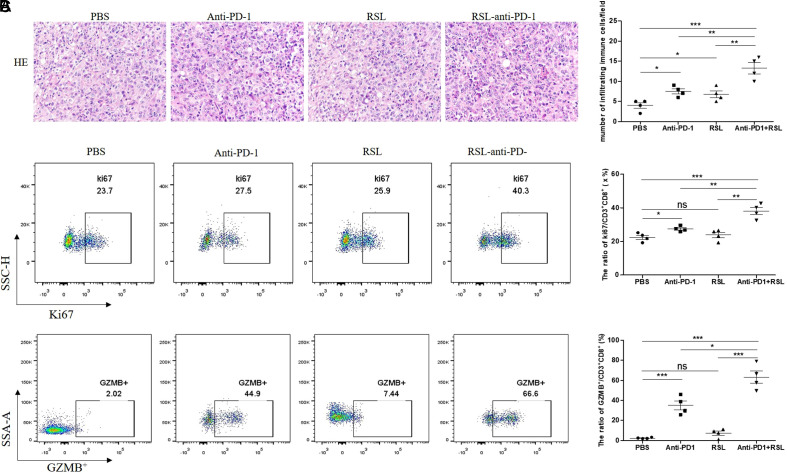
RSL may promote the antitumor effect of anti-PD-1 treatment by increasing the proliferation and cytotoxicity of CD8^+^ T cells. (A) HE staining of tumors in the PBS, anti-PD-1, RSL, and RSL+ anti-PD-1 treatment groups, n = 3. (B) Detection of the frequency of Ki67^+^ CD8^+^ T cells in the PBS, anti-PD-1, RSL, and RSL+ anti-PD-1 treatment groups, n = 4. (C) Detection of the frequency of GZMB^+^ CD8^+^ T cells in the PBS, anti-PD-1, RSL, and RSL+ anti-PD-1 treatment groups, n = 4. HE, hematoxylin and eosin; PD-1, programmed cell death protein 1.

To explore the underlying mechanism by which RSL promotes the antitumor effect of anti-PD-1 treatment in a syngeneic tumor mouse model, we analyzed the changes in immune cells in tumors from different groups. H&E staining demonstrated that both anti-PD-1 treatment and RSL treatment could obviously upregulate the infiltration of immune cells in tumors compared to that in the PBS group (Figure 5A). Importantly, the combination of anti-PD-1 and RSL could further statistically enhance the infiltration of immune cells compared to that in RSL or anti-PD-1 treatment alone (Figure 5A), suggesting that the synergetic antitumor effect of RSL and anti-PD-1 is correlated with the increasing infiltration of immune cells. Considering the key role of cytotoxic CD8^+^ T cells in the antitumor process, we measured the level of change in the frequency of GZMB^+^ CD8^+^ T cells. Our results showed that both RSL and PD-1 could slightly enhance the proliferation of CD8^+^ T cells, while the combination of RSL and PD-1 significantly increased the frequency of Ki67^+^ CD8^+^ T cells (Figure 5B), indicating the stronger proliferation of CD8^+^ T cells in tumors. Consistently, we also observed the highest increase in GZMB^+^ CD8^+^ T cells in the RSL+PD-1 treatment group (Figure 5B). Overall, RSL may promote the antitumor effect of anti-PD-1 treatment by increasing the proliferation and cytotoxicity of CD8^+^ T cells.

The strong release of tumor-derived neoantigens caused by RSL-induced ferroptosis may enhance the repertoire and frequency of cytotoxic IFN-γ^+^CD8^+^ T cells.

**Figure 6.**
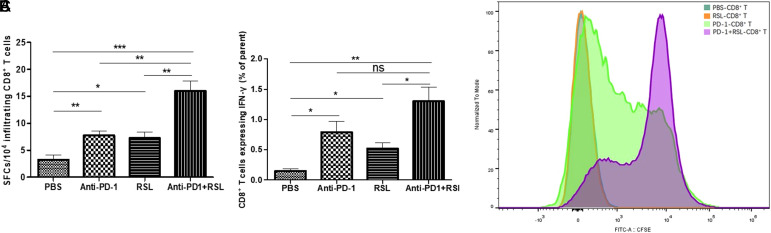
(A) RSL-induced ferroptosis may enhance the repertoire and frequency of cytotoxic IFN-γ^+^CD8^+^ T cells. (A) The frequency of SFCs in the PBS, anti-PD-1, RSL, and RSL+ anti-PD-1 treatment groups, n = 4. (B) The number of IFN-γ-positive infiltrating CD8^+^ T cells isolated from the PBS, anti-PD-1, RSL, and RSL+ anti-PD-1 treatment groups after coculture with MC-38 cells in vitro, n = 4. (C) The inhibition of infiltrating CD8^+^ T cells sorted from the tumors of the PBS, anti-PD-1, RSL, and RSL + anti-PD-1 treatment groups on tumor cell growth. Tumor cells were stained with CFSE, and the reduction rate of CFSE reflected the inhibition of CD8^+^ T cells on tumor cells. PD-1, programmed cell death protein 1.

To elucidate the mechanism by which RSL increases the proliferation and cytotoxicity of CD8^+^ T cells, we preliminarily analyzed the characteristics of CD8^+^ T cells in the 4 groups. As mentioned above, the accumulation of tumor-derived neoantigens after ferroptosis could be captured by MHC-I and recognized by the TCR on CD8^+^ T cells in the tumor microenvironment. Therefore, we isolated tumor-infiltrating CD8^+^ T cells and analyzed the frequency of tumor neoantigen-specific CD8^+^ T cells by IFN-γ ELISpot in the 4 groups. The neoantigen pool derived from tumor cell lysates treated with PBS, RSL, PD-1, or RSL + PD-1 was produced as described previously. After the in vitro stimulation of isolated tumor-infiltrating CD8^+^ T cells by the peptide pool, there was a significant increase in spot forming cells (SFCs) in both the RSL- and anti-PD-1-treated groups, while the combination of RSL and anti-PD-1 achieved the greatest percentage of SFCs (Figure 6A). Furthermore, RSL administration promoted the frequency of tumor neoantigen-specific IFN-γ^+^ CD8^+^ T cells in the PD-1 treatment groups (Figure 6B). In addition, the CD8^+^ T cells sorted from different groups were cocultured with CFSE-labeled MC-38 cells in vitro to detect the effect of RSL administration on cytotoxic capability. Consistently, the tumor-infiltrating CD8^+^ T cells isolated from RSL-treated tumors significantly inhibited the proliferation of MC-38 tumor cells compared with the CD8^+^ T cells isolated from the PBS, anti-PD-1 and RSL groups, indicating the higher cytotoxic capability of CD8^+^ T cells after RSL treatment (Figure 6C). Therefore, we concluded that RSL-induced ferroptosis may promote the antitumor effect of anti-PD-1 treatment by enhancing the repertoire and frequency of tumor neoantigen-specific IFN-γ^+^ CD8^+^ T cells.

## DISCUSSION

MC-38 is the third most common cancer and the second leading cause of cancer-related death worldwide.^25^ The difficulty of early screening, and the tendency to metastasize further augmented the bottleneck of CRC treatment.^26^ Drug 5-fluorouracil is the first line of chemotherapy in CRC, and many drugs, such as EGFR inhibitors, have also been investigated to block these processes in CRC. However, most patients develop severe resistance to the drug after long-term administration.^27^ After the successful application of PD-1/PD-L1-based immunotherapy in melanoma and lung cancer treatment, the development of immunotherapy for the treatment of CRC has aroused wide concern.^9,13^ However, the success of PD-1 inhibitors or CTLA-4 inhibitors is limited to only some subtypes of CRC. For example, the type of mismatch repair-proficient (pMMR)–MSI-L CRC constituted the large number of metastatic CRC (mCRC) cases and for which current immunotherapy strategies presented unsatisfying efficiency. Here, we investigated the effect of RSL-induced ferroptosis on the antitumor efficiency of anti-PD-1 treatment in a syngeneic CRC tumor mouse model and explored the underlying mechanism. We found that there was a positive correlation between the level of ferroptosis and the responsiveness of CRC to anti-PD-1 treatment in a mouse model. In addition, we found that RSL-induced ferroptosis may promote the antitumor efficiency of anti-PD-1 treatment by enhancing the repertoire and frequency of tumor neoantigen-specific IFN-γ^+^ CD8^+^ T cells.

The intratumoral immune microenvironment is immunosuppressive. To analyze the immune status of clinical CRC samples, we measured the expression levels of several crucial immune checkpoints, including PD-1, PD-L1, Tim3, TIGT, and CTLA-4. We found a significant increase in these immunosuppressive markers in tumors compared to those in paratumor tissues, which is consistent with previous studies reporting the accumulation of exhausted CD8^+^ T cells in CRC tumors.^28-30^ Notably, although PD-1 and PD-L1 expression presented obvious increases in all patients, not all of the patients were responsive to PD-1 treatment. In addition, Chen et al^31^ demonstrated that cytochrome P450 (CYP) 1B1 (CYP1B1)-derived 20-HETE promoted the ubiquitination and degradation of acyl-CoA synthetase long-chain family member 4 (ACSL4), ultimately inducing tumor cell resistance to ferroptosis, and the inhibition of CYP1B1 sensitized tumor cells to an anti-PD-1 antibody. To further elucidate the underlying mechanism, we divided the tumors into anti-PD-1-responsive and nonresponsive groups and analyzed the level of ferroptosis. Importantly, we found that tumors that were responsive to anti-PD-1 treatment exhibited a significant increase in ferroptosis compared to that of the nonresponsive group, indicating that the level of ferroptosis may affect the efficiency of PD-1 treatment in CRC. Previous evidence has shown that dysregulated ferroptosis-related genes play an important role in revealing intertumoral immune heterogeneity and indicate potential clinical benefits for anti–PD-1/PD-L1 immunotherapy in lung adenocarcinoma.^32^ Additionally, Zhou et al^32^ reported that TYRO3 inhibited tumor cell ferroptosis triggered by anti-PD-1/PD-L1 and facilitated the development of a protumor microenvironment by reducing the M1/M2 macrophage ratio, resulting in resistance to anti-PD-1/PD-L1 therapy. Inhibition of TYRO3 promoted tumor ferroptosis and sensitized resistant tumors to anti-PD-1 therapy. Therefore, the intervention of ferroptosis in tumors may regulate the antitumor efficacy of anti-PD-1 therapy in CRC.

To explore whether the intervention of ferroptosis by RSL could promote the efficiency of anti-PD-1 therapy, we analyzed the tumor growth of a subcutaneous syngeneic mouse model challenged by MC-38 cell lines. We found that RSL treatment could significantly increase ferroptosis in tumors and reduce the growth rate and weight of tumors in vivo, which is consistent with a previous study in a syngeneic 4T1 mouse model and in lung cancer patients.^33^ Therefore, RLS may be a promising drug to enhance the efficacy of anti-PD-1 treatment in CRC patients.

Our results showed that RSL-induced ferroptosis may promote the antitumor efficiency of anti-PD-1 treatment by enhancing the repertoire and frequency of tumor neoantigen-specific IFN-γ^+^ CD8^+^ T cells. There is evidence that ferroptosis appears to be more immunogenic due to the release of injury-associated molecular patterns, which in turn exacerbate the inflammatory response by releasing more tumor-specific neoantigens,^19^ which are subsequently loaded by MHC-I and capture TCRs to activate the tumor-specific toxicity of CD8^+^ T cells.^20,34,35^ In addition, Wang et al^21^ also demonstrated that anti-PD-1-based immunotherapy combined with ferroptosis induction was synergistic compared to monotherapy alone, indicating that ferroptosis induction may be an effective adjunct to anticancer immunotherapy. In our study, we found that both RSL and anti-PD-1 treatment could slightly enhance the proliferation of CD8^+^ T cells, while the combination of RSL and anti-PD-1 significantly increased the frequency of Ki67^+^CD8^+^ T cells, indicating the stronger proliferation of CD8^+^ T cells in tumors. Consistently, we also observed the highest increase in GZMB^+^ CD8^+^ T cells in the RSL + PD-1 treatment group. Therefore, we propose that the release of a large amount of tumor-derived neoantigens induced by RSL-mediated ferroptosis enhances the repertoire of tumor-specific CD8^+^ T cells and that anti-PD-1 treatment decreases the extent of exhaustion and promotes the proliferation of these tumor-specific CD8^+^ T cells to kill tumor cells.

In conclusion, we found that the level of ferroptosis is correlated with the antitumor efficacy of anti-PD-1 therapy, and RSL-induced ferroptosis promoted the efficiency of anti-PD-1 treatment in a syngeneic tumor model challenged by MC-38 cells. Mechanistically, RSL-induced ferroptosis enhanced the antitumor efficiency of anti-PD-1 treatment by increasing the repertoire and frequency of tumor neoantigen-specific CD8^+^ T cells.
